# Evaluating Nano‐Scale Shifts: Quantifying Mesopore Shrinkage in Porous Polymers via NMR Cryoporometry

**DOI:** 10.1002/smll.202500343

**Published:** 2025-04-03

**Authors:** Abdurrahman Bilican, Markus Leutzsch, Wolfgang Schmidt

**Affiliations:** ^1^ Max‐Planck‐Institut für Kohlenforschung Kaiser‐Wilhelm‐Platz 1 45470 Mülheim an der Ruhr Germany

**Keywords:** pore size determination, resorcinol‐formaldehyde gel

## Abstract

Nanoporous materials synthesized in a wet‐chemical process often show different pore sizes in their as‐prepared state in the reaction solution and their dried state. Depending on the materials, these changes can be reversible or irreversible. Determination of the exact pore sizes of such materials in their as‐prepared state is challenging as conventional characterization methods for pore size determination only work on dry materials. Cryoporometry is applicable directly to wet materials though. Thus, it offers a unique opportunity for analyzing unaffected pores of materials prior to drying. With this method the “true” size distributions of pores in wet materials are accessible. Shrinkage of gels upon ambient drying is a known phenomenon. However, exact numbers are not accessible even though being essential for process design. Here, nuclear magnetic resonance (NMR) cryoporometry is used for determining pore sizes of sol‐gel derived gels before and after drying, allowing for quantitative assessment of drying‐induced pore shrinkage, an immensely valuable information barely accessible with other methods. The magnitude of pore shrinkage in the gels depends strongly on synthesis parameters. Higher monomer concentrations can mitigate drying‐induced pore shrinkage. The quantitative information obtained with cryoporometry will allow for optimization and efficient design of porous materials for various applications.

## Graphical abstract

NMR cryoporometry allows for the determination of nanopore diameters in wet polymer gels. Pore shrinkage upon thermal drying of the gels is precisely gauched and related to parameters used for the synthesis of polymer gel. The data show that sufficiently high monomer concentrations can completely mitigate pore size reduction upon drying.

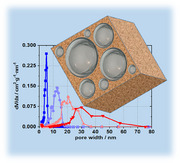

## 1 Introduction

Porous materials play an important role in the transformation toward a sustainable economy in our modern society. Their design and optimization are crucial in the fields of catalysis, energy storage, and adsorption technology. Porous solids can be obtained by a variety of synthetic routes, one of which is sol‐gel synthesis, where a porous solid in the form of a gel is formed from a reaction solution. A major challenge is then the removal of occluded solvent from the pores of the obtained gel. Due to the small pores and the solvent/air surface tension, high capillary pressures build up as the liquid leaves the pores, resulting in the shrinkage of the gel material to compensate for the internal stress generated by the capillary pressure.^[^ ^1^ ^]^ To overcome these effects, supercritical or freeze drying is used as these methods avoid the generation of high capillary pressure. Supercritical drying and freeze‐drying are energy‐intensive and require additional production steps, which has a negative impact on the economics of large‐scale production. Therefore, attention is focused on xerogels, which include a simple ambient drying step, making the production of porous solids via a sol‐gel route more industrially relevant. However, ambient drying often has the disadvantage of significant shrinkage of the porous material. The scope of material design within these constraints lies in optimizing the synthesis parameters to obtain the desired porous properties. One of the critical properties is the pore size distribution, which is usually unknown prior to the drying step. As the pore size determination via the conventional sorption methods requires porous materials with empty pores, removal of the liquid from the pores prior to the measurement is essential. This so‐called activation step can go along with substantial shrinkage of the pores though and pore sizes derived with that method do not necessarily reflect those in as‐synthesized porous materials. In this study, we present an approach to determine the pore size distribution of porous polymers in their as‐synthesized state by NMR cryoporometry.^[^ ^2^ ^]^ NMR cryoporometry, the method used here, is a technique used to study phase transitions in materials confined within pores. The method primarily focuses on the solid‐liquid transitions and interprets shifts in melting and freezing points to determine pore sizes, pore size distributions, and, occasionally, pore shapes.^[^ ^3^ ^]^ The Gibbs–Thomson equation is central to this technique, relating the melting point depression (*ΔT_m_*) of ice melting in nanoscopic pores to the diameter (*x*) of the solid ice phase.^[^ ^2^, ^4^ ^]^ As the ice is confined within the pores, the diameter of the solid ice phase (*x*) reflects the pore diameter.^[^ ^5^ ^]^ While the Gibbs–Thomson equation usually assumes either spherical or cylindrical pores, real materials often show more complex pore geometries.^[^ ^4a^ ^]^ Using the cylindrical pore model has become a frequent choice for such materials. NMR cryoporometry leverages the sensitivity of NMR signals to molecular motion, distinguishing between the broad signals of solids and the narrower signals of liquids. Spin‐echo sequences, such as the Hahn echo and Carr‐Purcell‐Meiboom‐Gill (CPMG), are used to filter out signals from solids, based on their shorter T_2_ relaxation times, allowing focus on the liquid phase.^[^ ^6^ ^]^ The NMR signal intensity varies with temperature, reflecting the fraction of molten water within the pores. By assuming a relationship between signal intensity and pore volume, a pore‐size distribution can be obtained, though it is typically relative without absolute scaling.^[^ ^4b^ ^]^ Temperature affects signal intensity through Curie's law, probe electronics, and molecular dynamics, complicating data interpretation. Normalizing with a non‐freezing liquid or adjusting the relaxation delay can mitigate these issues.^[^ ^7^ ^]^ NMR cryoporometry has been applied to various materials, including porous polymers in aqueous environments, which are challenging to study with conventional techniques. Petrov et al. studied the evolution of pore structure in biodegradable polymer microparticles, revealing correlations between pore size and drug release rates.^[^ ^8^ ^]^ Similarly, Hansen et al. investigated polystyrene cross‐linked with divinylbenzene in water, identifying mean pore radii.^[^ ^9^ ^]^ Other applications of the method include studies on bone, coal, paper, and polyelectrolyte multilayers (PEMs).^[^ ^10^ ^]^ In summary, NMR cryoporometry is a powerful tool for characterizing porous materials, offering insights into pore structure and its impact on material properties, though careful consideration of its limitations is essential. In this work, the method is used for studying the fundamentals of the formation of porous resorcinol‐formaldehyde (RF) gels within reaction solutions and quantifying the effect of relative shrinkage on pores upon solvent removal. RF gels are known to be subject to an irreversible decrease of pore volume upon solvent removal under sub‐critical conditions. This reduction in pore volume is accompanied by a noticeable decrease in pore size, underscoring the profound impact of drying techniques on the final gel structure.^[^ ^11^ ^]^ Despite the use of advanced drying methodologies, such as cryo drying or supercritical drying, which aim to preserve the gel's porous architecture, a certain degree of shrinkage in the RF monoliths is usually unavoidable.^[^ ^11c^ ^]^ The shrinkage leads to significant changes or the porous properties of RF gels, which poses substantial problems for analyzing the porous properties of as‐synthesized RF gels as most common characterization methods require pre‐drying of samples. In that context, Yamamoto et al. employed thermoporometry via differential scanning calorimetry (DSC) for analyzing RF hydrogels in their as‐prepared state.^[^ ^12^ ^]^ However, up to now, no quantitative data exist to describe the degree of pore size shrinkage in an unbiased manner. Here, cryoporometry provides the unique opportunity to investigate RF gels in their as‐synthesized and dried state using the same methodology.

## 2 Results

RF gels exist in two distinct states, i.e., in their as‐synthesized state denoted as RF hydrogels to address their water‐containing state prior to drying. It must be emphasized that the RF gels are not hydrogels in the sense that they can swell and shrink upon solvent uptake or release. Drying causes irreversible shrinkage of the gel. After drying, the materials are then denoted as RF xerogels. The sample code assigns hydrogels (H) and xerogels (X). Further, it provides information on the resorcinol to catalyst ratio (RC) and the total mass fraction of resorcinol and formaldehyde (M%) in the reaction mixture as RC/M%. Generally, higher RC values tend to cause larger pores, whereas higher M% (higher concentration of monomers) results in smaller pores and narrower pore size distributions. For the evaluation of pore size distributions, the Gibbs–Thomson equation (Equation 1) describes the depression of the melting temperature *ΔT_m_* of a frozen fluid confined in pores as a function of the size *x* of the frozen particle and a number of constants, *T_m_* being the bulk melting temperature of the fluid.^[^ ^13^ ^]^

(1) $$\Delta T_{m}=T_{m}\;-T_{m}\left(x\right)=\frac{4\gamma_{\mathrm{sl}}T_{m}}{x\Delta H_{f}\rho_{s}}\;$$

While the density of the solid *ρ_S_* can be obtained by pycnometry, the surface energy at the solid‐liquid interface *γ_sl_*, and the bulk enthalpy of fusion *ΔH_f_*, cannot be determined easily. Therefore, Hansen et al. proposed a simplification of Equation (2) in which the constants in Equation (1) are combined to the constant *k_c_*, and the surface layer thickness of the unfrozen pore water *2sl*, can be determined empirically using the pore width values obtained from the BJH analysis.^[^ ^14^ ^]^ According to Equation (3), plotting the BJH pore width *x* versus the inverse melting temperature depression *ΔT_m_^−1^* of each sample yields a linear relationship where *k_c_* and *2sl* can be determined by linear fitting.^[^ ^2^, ^15^ ^]^

(2) $$\Delta T_{m}=\frac{k_{c}}{x-2sl}\;$$

(3) $$x=\frac{k_{c}}{\Delta T_{m}}+2sl$$

With the obtained data, the normalized pore size distribution *dv/dx* can be obtained from the first derivative *dv/dT_M_* of the melting curve using Equation (4) proposed by Strange et al.^[^ ^4b^ ^]^

(4) $$\frac{dv}{dx}=\frac{k_{c}}{x^{2}}\;\cdot \frac{dv}{dT_{m}}$$

Using the pore size distribution, the mode value of the pore width is obtained for hydrogels and xerogels, and the shrinkage is calculated using Equation (5).

(5) $$\varepsilon =\frac{x_{\mathrm{hydr}\mathrm{ogel}}-x_{\mathrm{xero}\mathrm{gel}}}{x_{\mathrm{hydr}\mathrm{ogel}}}\;$$

**Figure 1:**
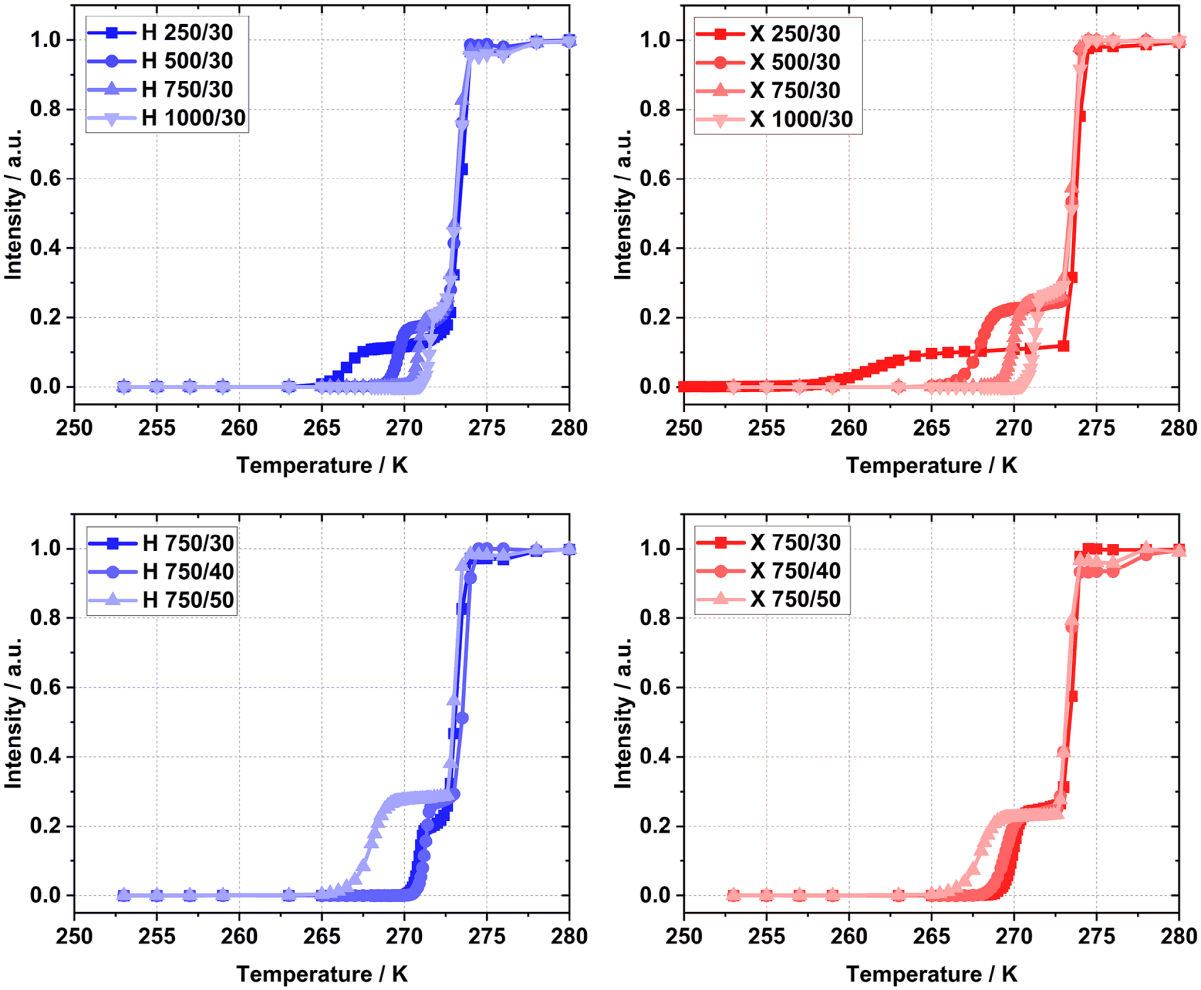
NMR melting curves of water in RF gels before (blue) and after drying (red).

The spin echo ^1^H NMR measurements were subjected to analysis, resulting in the generation of the melting temperature curves depicted in **Figure** **1**. These curves illustrate the occurrence of distinct stages of melting with increasing temperature. At distinct temperatures, the signal begins to increase as the water within the pores commences to melt. The melting continues until the first plateau is reached, which represents the total pore volume.

The following rapid increase in signal intensity at higher temperatures indicates the onset of bulk water melting, which subsequently reaches the second plateau, signifying the total liquid volume.^[^ ^16^ ^]^ As illustrated in Figure 1, the melting temperature curves demonstrate that an elevated *RC* value correlates with the onset of water melting in the pores at higher temperatures. This suggests that the pore sizes are larger with increasing *RC* value, which is consistent with the N_2_ sorption data (Figures S1 and S2, Supporting Information) and with findings reported in the literature. A comparison of the melting curves from the hydrogels and xerogels reveals a notable alteration in their profiles. While the melting curve of sample X 1000/30 only exhibits a slight shift toward lower temperatures, the change becomes more pronounced with a reduction in *RC* value. Sample X 250/30 exhibits a wider melting temperature range, a phenomenon also observed by Rottreau et al. in porous silica with small pore sizes. It is assumed that the tortuous pore system contains residual water which diffuses within the interconnected pores.^[^ ^17^ ^]^ The impact of elevated monomer concentrations in sol‐gel reaction solutions has been extensively investigated and is widely recognized to result in the formation of narrower and smaller pore size distributions.^[^ ^18^ ^]^ However, the densification of the solution to *M%* = 40 did not elicit a notable shift of the melting temperature curve toward lower temperatures. Only at *M%* = 50 a discernible shift became evident.

**Figure 2:**
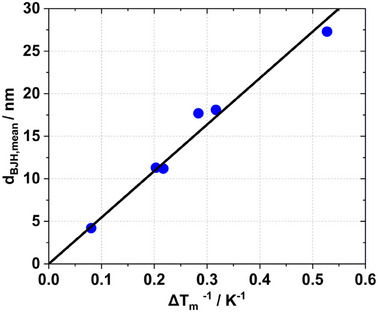
Correlation between the mean pore size of RF xerogels derived from N_2_ sorption isotherms and the mean inverse melting point depression *ΔT_M_^−1^*.

The parameters *k_c_* and *2sl* were calculated from the slope and y‐intercept in Equation (3) by plotting the calculated BJH pore widths (*d_BJH,mode_*) from N_2_ sorption analyses of the obtained RF xerogels against the inverse melting temperature depression *ΔT_M_^−1^* (Table S1, Supporting Information). **Figure** **2** shows a linear correlation between these two parameters. The thickness of the non‐freezing pore water layer depends on the pore size, shape, and bulk material of the porous medium.^[^ ^12^ ^]^ Fitting the data resulted in *k_c_* of 52.3 K·nm and *2sl* of 0.78 nm. Thus, the surface layer would have a thickness of 0.39 nm, which is about the thickness of one to two layers of water. Since the focus of this study was to compare the pore size distribution of non‐shrunk and shrunk mesoporous polymers, the model was simplified by fitting Equation (3) with a fixed *2sl* parameter of 0 nm which resulted in *k_c_* of 54.6 ± 1.7 K·nm The error introduced by this is relatively small as illustrated in Figure S3 (Supporting Information). In any case, data from NMR cryoporometry using the Gibbs–Thomson equation will allow a direct evaluation of pore shrinkage upon drying of RF gels. Systematic errors, if existing, should be more or less the same for all samples and thus allow for a direct comparison of data.

**Figure 3:**
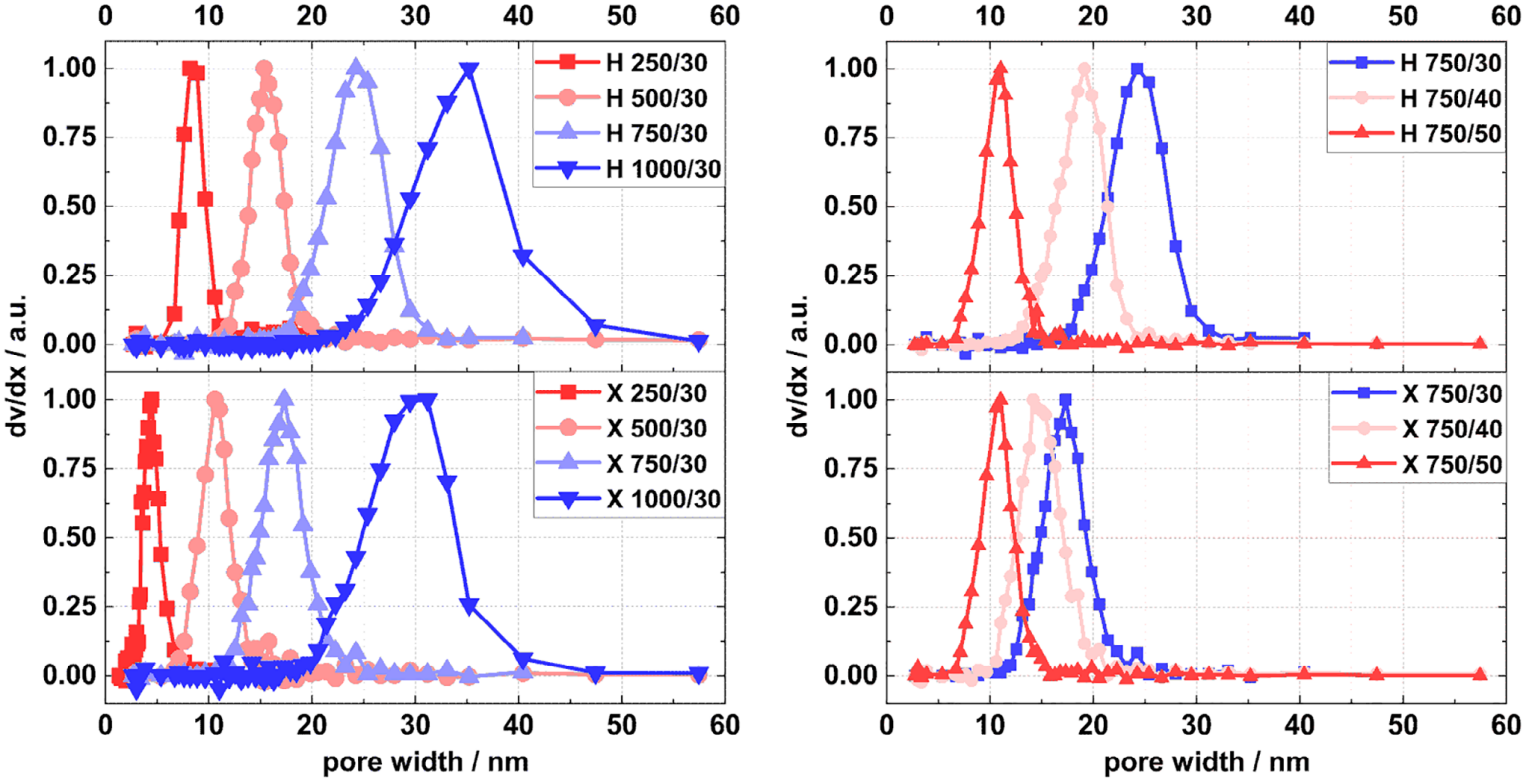
Pore size distributions of hydrogels (H) and xerogels (X) synthesized with varied *RC* (left) and with varied *M%* (right) values derived from NMR melting curves. The curves were normalized for easier comparison.

For the calculation of pore size distributions of the wet and dried RF gels with Equation (5), the first derivatives (*dv/dT_M_*) of the melting curves in Figure 1 need to be transferred into pore size distributions (*dv/dx*) using Equation (3). The resulting pore size distributions for the as‐prepared RF hydrogels and dried RF xerogels are presented in **Figure** **3**.

The upper sections of the graphs present the pore size distributions for the hydrogels, while the lower sections show the ones for the xerogels. The graph shows the effect of changing *RC* and fixed *M%* value on the left and that of the effect of changing *M%* and fixed *RC* value on the right. Wet and dried gel with the same synthesis parameters are consistently represented by the same color code. **Table** **1** lists the mean pore sizes for the hydrogels *x_Hydrogel_* and xerogels *x_Xerogel_* as derived from the data shown in Figure 3. With constant *M%* and increasing RC, generally larger pores are generated for both RF hydrogels and dried RF xerogels. On the contrary, the pore sizes of RF hydrogels and RF xerogels get reduced with increasing M% and fixed *RC*. Direct comparison of corresponding wet and dry gels reveals significant shrinkage of pores upon drying though. This observation holds for all RF gel pairs except for the pair 750/50, which shows no pore size reduction upon drying.

**Table 1:** Mean pore sizes of RF xerogels derived from N_2_ sorption (*d_Xerogel,BJH_*) and of hydrogels (*x_Hydrogel_*) and xerogels (*x_Xerogel_*) derived from NMR cryoporometry and their relative pore size reduction ε after drying.

sample	*d_Xerogel,BJH_*/nm	*x_Hydrogel_*/nm	*x_Xerogel_*/nm	*ε*/%
250/30	4.2	9.7	4.2	57
500/30	11.3	16.0	8.5	47
750/30	18.1	23.6	17.0	28
1000/30	27.3	32.2	28.1	13
750/40	17.7	18.4	15.2	17
750/50	11.2	11.1	11.1	0

For easier assessment of the pore shrinkage upon drying, Table 1 lists the relative pore size reduction parameter *(ε)* as calculated with Equation (5). The pore shrinkage shows a clear trend with both increasing *RC* and increasing *M%*. For hydrogels with a constant monomer density (M%), the relative reduction in pore size is more pronounced in samples with smaller initial pore sizes (lower RC values). This phenomenon can be attributed to the scaling of capillary forces with the inverse of the pore radius.^[^ ^19^ ^]^ Consequently, smaller pores experience higher capillary forces, leading to a stronger relative reduction in pore size. When the monomer density (M%) is increased while maintaining a constant pore size (RC), the expected trend based on capillary forces does not hold. Specifically, the relative shrinkage of pore size decreases with higher M%, despite the smaller original pore size. The increase in monomer density enhances the stability of the gel structure. This increased stability compensates for the higher capillary forces, thereby reducing the extent of pore size shrinkage. The pore size reduction *(ε)* becomes zero for *RC* = 750 and *M%* = 50. H 750/50 obviously has a very firm gel structure, stable enough to withstand shrinkage during thermal drying.

Léonard et al. report lower shrinkage for RF gels synthesized with a higher *RC* value by measuring the total volume of a porous monolith before and after ambient drying.^[^ ^20^ ^]^ Job et al. showed that higher levels of reactants in the solution generally reduce the overall volumetric shrinkage of RF gel monoliths.^[^ ^11c^ ^]^ Our study confirms these findings and provides detailed information on the change of pore size and distribution associated with volumetric shrinkage upon drying. The shown method of measuring NMR croyporometry of as‐prepared and thermally dried samples reveals precisely the degree of pore size reduction upon drying.

**Figure 4:**
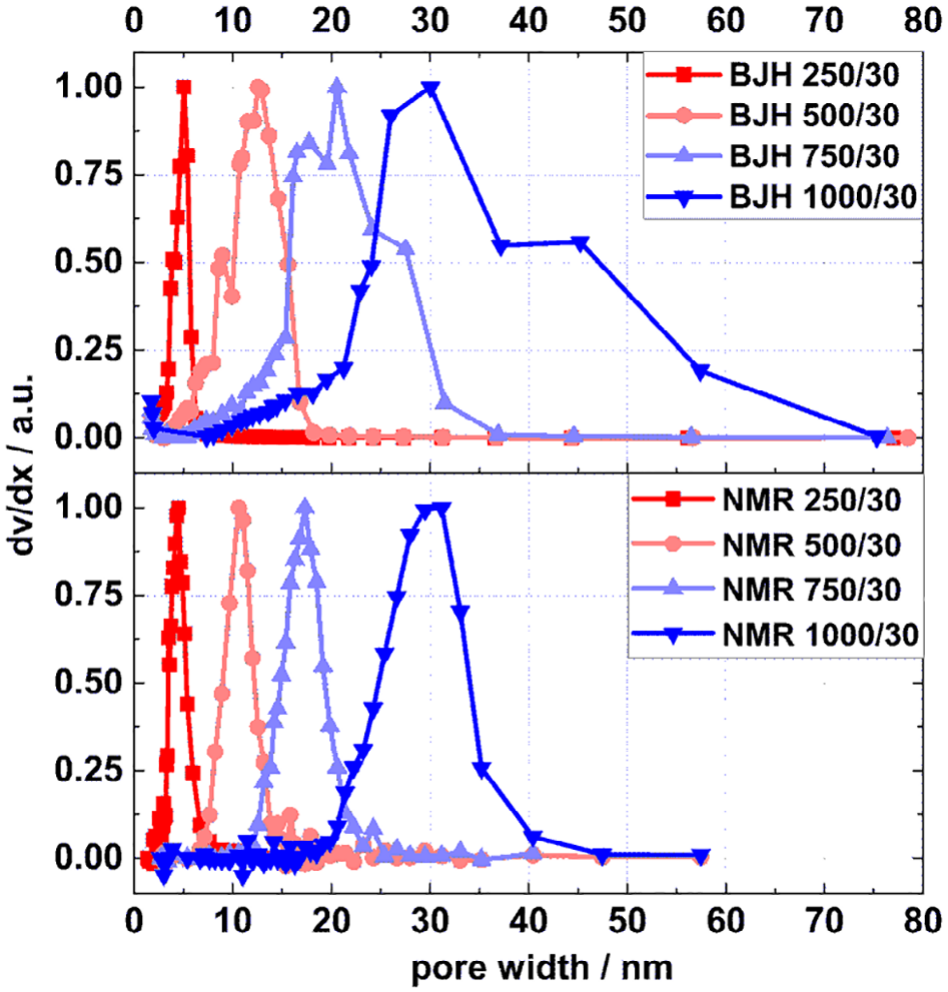
Pore size distribution of RF xerogels (RF = 250 – 1000) derived from N_2_ sorption (top) and NMR cryoporometry (bottom).

The mean pore sizes of xerogels obtained from both the BJH (*d_Xerogel BJH_*) and Gibbs–Thomson (x_Xerogel_) methods are listed in Table 1. Pore sizes derived from BJH and NMR for the RF xerogels differ to some extent. Certain differences are also reflected in the shapes of the pores size distributions obtained from both methods. **Figure** **4** illustrates that pore size distributions derived from NMR cryoporometry tend to be narrower than those derived from N₂ sorption isotherms. The Kelvin equation, as used in the BJH model, and the Gibbs–Thomson equation are widely analogous.^[^ ^5b^ ^]^ In the case of the Kelvin equation, a curved liquid surface is in equilibrium with a volatile vapor or gas phase, in the case of the Gibbs–Thomson equation, a nanoscopic solid phase (ice) is in equilibrium with a large volume of a surrounding liquid phase. For the Kelvin equation, the effect of curvature of a surface is decisive, for the Gibbs–Thomson equation it is the effect of size of the specific surface area (particle size).^[^ ^5b^ ^]^ It is thus not surprising that pore size distributions derived with the two methods differ to some extent as they are based on two different phase transitions, i.e., gas adsorption/desorption from a liquid on the one hand and melting/solidification of a solid phase in a liquid on the other. The fact that we did not determine an extended liquid phase but only one or two layers of liquid water around the ice crystals might also contribute to differences, as such a thin film is not fully in line with the Gibbs–Thomson model (assuming a large liquid volume). Thus, some differences exist, but in general, the data derived from both methods are well comparable. The remaining consideration is the relative strengths and weaknesses of the two analytical methods. NMR cryoporometry is a non‐invasive technique which permits determination of pore size distributions not only from dried solids but also from solids immersed in liquids. Of course, determination of unknown parameters is essential, for which a second method, such as N_2_ sorption analysis, is indispensable. Using different solvents with different surface interactions and melting temperatures, an optimized experiment protocol can be designed for each material and pore size. When it comes to the assessment of pore sizes and pore volume, N_2_ sorption is a more convenient and straight‐forward method. In addition, sorption instruments are more abundant than NMR spectrometers and available in most laboratories working on porous solids. On the contrary, cryoporometry is also possible with convenient cryo‐DSC instruments.

## 3 Conclusion

NMR cryoporometry was performed on as‐prepared and dried hydrothermally synthesized, mesoporous RF gels using simple spin echo ^1^H NMR measurements. The obtained melting curves were transformed into pore size distributions using the Gibbs‐Thomson equation. This allowed the evaluation of pore size distributions of sol‐gel derived porous materials in their original wet state without being affected by material shrinkage. Comparison of pore sizes of wet RF gels and dried RF gels allowed direct quantification of pore shrinkage upon drying of the wet gels. Depending on *RC* and *M%* used for the synthesis, shrinkage can be substantial, for low *RC* and *M%*, or completely absent, as for RF gels obtained with *RC* = 750 and *M%* = 50. The data above show that cryoporometry provides the unique option to analyze porous gel structures in their wet and as‐prepared state. Using this method, pore shrinkage of gel structures can be precisely monitored which strongly will enhance the understanding of drying processes in sol‐gel chemistry and allow for a rational material design of sol‐gel derived materials.

## 4 Experimental Section

### Synthesis

Resorcinol (ACS reagent ≥ 99%, Sigma Aldrich) was dissolved in a 37 wt.% aqueous formaldehyde solution (ACS reagent, 36.5‐38.0%, stab. with 10–15% methanol) at a molar resorcinol/formaldehyde ratio (R/F) of 1:2. An appropriate amount of water was then added to give the desired *M%*.

(6) $$M\%\;=\frac{m_{Resorcinol}+m_{Formaldehyde}}{m_{total}}$$

For the synthesis of resorcinol‐formaldehyde (RF) gels with different porous properties, varying amounts of 0.05 m aqueous solution of Na_2_CO_3_ (Titripur, Merck KGaA Germany) were used to obtain the required *RC* values. The RC values were varied at 250, 500, 750, and 1000 at *M%* = 30%.

(7) $$RC=\frac{n_{Resorcinol}}{n_{Na2CO3}}$$

For investigating the influence of *M%* on the porous properties, RF gels with a fixed RC value of 750 and a varied *M%* of 30%, 40%, and 50% were prepared.

The solutions were stirred briefly and then poured into Teflon liners which were placed in steel autoclaves. The autoclaves were placed in a heating block at 120 °C for 60 min. One part of each solidified RF gel was then soaked into mQ Water to form a hydrogel. To minimize the remaining reaction solvent inside the pores, the water exchange was performed over a period of 3 days by daily replacing the supernatant liquid with fresh mQ water. The other part of the obtained gels were dried at 80 °C for 24 h to form a xerogel. Hydrogels were denoted as H *RC/M%* and xerogels as X *RC/M%*.

### N_2_ Sorption

For N_2_ sorption analyses at liquid nitrogen temperature, the RF xerogels were degassed under vacuum for 12 h at 80 °C prior to sorption measurements on a Micrometrics 3Flex instrument. The MicroActive software provided by Micromeritics was used for data analysis. The Brunauer–Emmet–Teller (BET) theory was used to calculate the apparent specific surface areas, *S_BET_*. The evaluation of the BET surface area was performed according to the Rouquerol criteria. For the calculation of micropore volumes, *V_mic_*, and external specific surface areas, *S_ext_*, the t‐plot method was used, applying the equation of Harkins and Jura for the calculation of the thickness of the adsorbed layer inside the pores. The pore width (mode), *d*_pore_, in the meso‐ and macropore range was analyzed by the Barret‐Joyner‐Halenda (BJH) method using the desorption branches of the isotherms.^[^ ^21^ ^]^

### NMR Cryoporometry

Samples were prepared by wetting the coarse gel particles with mQ water and grinding them to a wet powder. The powder was then soaked in mQ water for 24 h to obtain a fine dispersion, which was filled into 5 mm screw cap NMR tubes. Cryoporometry measurements were conducted on a Bruker AVANCE IIIHD 400 MHz NMR spectrometer equipped with a 5 mm BBFO probe with z‐gradient and a Bruker BCU II cooling unit. Temperatures in the range from 283 to 243 K were calibrated with a solution of 4% MeOH in MeOD using the Bruker Topspin AU program *calctemp*. ^1^H NMR spectra were acquired to measure the relationship between the reading of the temperature sensor and the temperature of the reference sample at different temperatures. Then linear temperature correction factors were obtained from a linear regression after plotting the set point temperatures versus the measured temperatures. These correction factors were then used in the temperature control unit of the spectrometer during the measurement. For the variable temperature measurements, a N_2_ flow rate of 800 L·h^−1^ and the setting *strong* (level 3) of the cooling unit were used.

Melting curves were obtained by freezing the in‐pore liquid until the NMR signal completely vanished and then heating the sample in small temperature steps and collecting NMR signals for each temperature until complete liquefaction (melting) of the in‐pore and bulk liquid. At each temperature, the sample was kept for 10 min to stabilize the temperature. Then the probe was automatically tuned and matched. Afterward, ^1^H NMR data was acquired with a *T_2_* filter (single spin echo with an echo time of 2 ms, Bruker sequence: cpmg1d, *d_20_* = 1 ms) to suppress residual signal from the solid. These acquisition parameters were previously described by Rotterau et al.^[^ ^17^ ^]^ The acquired NMR data was imported into Mestrelab MNOVA 15.0.0 with the *Reaction Monitoring* plugin and processed therein (Fourier transformation, phasing, baseline correction, integration). Generally, only a zero order phasing was used and the baseline was corrected using a zero order polynomial. The signal of the water was integrated ± 15 ppm around the maximum peak.

## Conflict of Interest

The authors declare no conflict of interest.

## Supporting information



Supporting Information

## Data Availability

The data that support the findings of this study are available in the supplementary material of this article.
