# Supplementary material for: Evaluating Nano‐Scale Shifts: Quantifying Mesopore Shrinkage in Porous Polymers via NMR Cryoporometry
Source: Small. 2025 Apr 3;21(21):2500343. doi: 10.1002/smll.202500343 (PMC12105444; doi:10.1002/smll.202500343)
Supplement: Supplementary file 1 — Supporting Information [file SMLL-21-2500343-s001.docx]

**Evaluating Nano-scale Shifts: Quantifying Mesopore Shrinkage in Porous Polymers via NMR Cryoporometry**

Abdurrahman Bilican,^a^ Markus Leutzsch,*^,a,^ Wolfgang Schmidt *^,a^

^a^ Max-Planck-Institut für Kohlenforschung, Kaiser-Wilhelm-Platz 1, 45470 Mülheim an der Ruhr, Germany

E-mail: leutzsch@mpi-muelheim.mpg.de, schmidt@mpi-muelheim.mpg.de

Supporting Information

**Figure S1.** N_2_ sorption isotherms of RF xerogels.

**Figure S2.** BJH pore size distribution of RF xerogels.

**Table S1.** Pore sizes *d_x,BJH_* of RF xerogels derived from N_2_ sorption and melting temperature *T_M_* derived from NMR cryoporometry and the respective inverse relative melting temperature depression *ΔT_M_^-1^*.

| **sample** | ***d_x,BJH_*  nm** | ***T_M_*  K** | ***ΔT_M_^-1^* K^-1^** |
| --- | --- | --- | --- |
| **X 250/30** | 4.2 | 260.7 | 0.080 |
| **X 500/30** | 11.3 | 268.2 | 0.203 |
| **X 750/30** | 18.1 | 270.0 | 0.317 |
| **X 1000/30** | 27.3 | 271.3 | 0.527 |
| **X 750/40** | 17.7 | 269.6 | 0.284 |
| **X 750/50** | 11.2 | 268.5 | 0.217 |

**Figure S3.** Comparison of pore size distributions calculated by either using 2sl values from fitting (denoted by appendix k_sl_) or by setting 2sl = 0 (denoted by appendix k_0_).
